# Unveiling Subtle Geographical Clines: Phenotypic Effects and Dynamics of Circadian Clock Gene Polymorphisms

**DOI:** 10.3390/biology12060858

**Published:** 2023-06-14

**Authors:** Loren Khatib, Bengisu Sezen Subasi, Bettina Fishman, Martin Kapun, Eran Tauber

**Affiliations:** 1 Department of Evolutionary and Environmental Biology, Institute of Evolution, University of Haifa, Haifa 3498838, Israel; 2 Natural History Museum Vienna, 1010 Vienna, Austria; 3 Department of Cell and Developmental Biology, Medical University of Vienna, 1090 Vienna, Austria

**Keywords:** circadian clock, latitudinal cline, molecular polymorphism, adaptation

## Abstract

**Simple Summary:**

Circadian clocks are molecular pacemakers that drive daily rhythms in physiology, metabolism, and behavior. They are present in a diverse range of organisms, from cyanobacteria to humans. In the last few decades, enormous progress has been made in understanding the molecular details of the circadian clock. The fruit-fly *Drosophila* has been instrumental in identifying ‘clock’ genes, which are well conserved in mammals, both in sequence and function. Many ‘clock’ genes have been identified using laboratory mutagenesis screens, which look for mutants that show aberrant circadian behavior. The recent accumulation of genomic sequences from wild populations of *Drosophila* provides an opportunity to identify natural genetic variations in ‘clock’ genes, which serve as molecular adaptations to local habitats. Here, we focused on nine polymorphic sites in genes associated with the clock and tested their impact on diurnal behavior. We found seven sites whose alleles confer different circadian behavior or seasonal response. This analysis of natural variation can provide information about fine-tuning of the clock, and it is essential for understanding the evolution of the circadian system.

**Abstract:**

Our understanding of the gene regulatory network that constitutes the circadian clock has greatly increased in recent decades, notably due to the use of *Drosophila* as a model system. In contrast, the analysis of natural genetic variation that enables the robust function of the clock under a broad range of environments has developed more slowly. In the current study, we analyzed comprehensive genome sequencing data from wild European populations of *Drosophila*, which were densely sampled through time and space. We identified hundreds of single nucleotide polymorphisms (SNPs) in nine genes associated with the clock, 276 of which exhibited a latitudinal cline in their allele frequencies. While the effect sizes of these clinal patterns were small, indicating subtle adaptations driven by natural selection, they provided important insights into the genetic dynamics of circadian rhythms in natural populations. We selected nine SNPs in different genes and assessed their impact on circadian and seasonal phenotypes by reconstructing outbred populations fixed for either of the SNP alleles, from inbred DGRP strains. The circadian free-running period of the locomotor activity rhythm was affected by an SNP in *doubletime* (dbt) and *eyes absent* (*Eya*). The SNPs in *Clock* (*Clk*), *Shaggy* (*Sgg*), *period* (*per*), and *timeless* (*tim*) affected the acrophase. The alleles of the SNP in *Eya* conferred different levels of diapause and the chill coma recovery response.

## 1. Introduction

The circadian clock is an evolutionarily conserved system, which is present in a broad range of organisms, from cyanobacteria to humans. The circadian clock drives a large number of physiological and cellular diurnal rhythms. The ubiquity of the circadian clock alludes to its importance to fitness: having a functional clock allows organisms to predict rhythmic environmental changes, and consequently improve their fitness [1].

The molecular circuit underlying the circadian system has been extensively investigated, and studies on both insects and rodents revealed that it is based on a transcription-translation negative feedback loop (TTFL) [2]. In *Drosophila*, the transcription of clock genes *period* (*per*) and *timeless* (*tim*), which starts at midday, is driven by two transcription factors: Clock (CLK) and Cycle (CYC). These two proteins form a dimer that binds to E-box enhancers in the *per* and *tim* promoters. Transcripts of *per* and *tim* accumulate in the cytoplasm, and their protein products form a dimer that translocates back to the nucleus, where it binds to CLK/CYC and inhibits their own transcription. This inhibition lasts until the expression of *per* and *tim* is reduced, and the whole cycle starts again [3]. The pace of the cycle is modulated by several kinases that phosphorylate PER and TIM. Two examples are DOUBLETIME (DBT) and SHAGGY (SGG), which regulate the stability of PER and TIM and their nuclear entry. In addition to the PER/TIM loop, another interlocked loop exists, in which Vrille (VRI) represses *Clk* transcription, while PAR-domain protein 1ε (PDP1ε) activates it [4]. The output of the circadian oscillator is relayed by output proteins such as the neuropeptide pigment-dispersing factor (PDF) and its receptor (PDFR).

Fluctuations in the environment, particularly in temperature, present a significant challenge to the circadian system, which is required to generate stable 24 h rhythms. One may expect that wild populations in various geographical regions will evolve molecular variations that serve as local adaptations to different environmental conditions. *Drosophila*, which has been a major model system in population genetics since the pioneering work of Dobzhanski [5], became a natural choice for studying the evolution and adaptation of the circadian system.

The study of the spatial pattern of genetic variation in wild populations is particularly informative for studying adaptation. When genetic or phenotypic variation changes gradually across a geographical gradient, an adaptive significance of that variation is generally assumed and is considered the signature of natural selection. Latitudinal clines (where allele frequency changes systematically with latitude) are a classic example of the signature of natural selection and were studied in numerous genes in *Drosophila* [6]. Latitudinal clines were also found in different clock genes [7]. For example, analysis of *per* gene alleles in *D. melanogaster* natural populations in Europe revealed a length polymorphism in the Thr-Gly region. Two major alleles, (Thr-Gly)^17^ and (Thr-Gly)^20^, accounted for 90% of the variation, *per* (Thr-Gly)^23^ making up 8% and *per* (Thr-Gly)^14^ at 1%. Importantly, there was a significant difference in their geographic pattern, with (Thr-Gly)^20^ being more abundant in Northern Europe, whereas (Thr-Gly)^17^ was more frequent in the south, resulting in a robust latitudinal cline. The *per* polymorphism was shown to be important for the thermal adaptation of flies in different environments [8].

A latitude cline was also found in *tim* [9]. A single nucleotide insertion/deletion in the 5’ coding region defines two alleles (*ls*-*tim*/*s*-*tim*) that segregate in wild populations, with *ls*-*tim* being most frequent in southeastern Europe, whereas *s*-*tim* prevails in the north. This polymorphism was shown to be driven by a directional selection that favors the new allele (*ls-tim*), which is more cold-adaptive. The clinal pattern in this polymorphism reflects the recent spread of the new allele from the south of Italy and represents an evolutionary transient. A recent study [10] revealed that under continuous light (or extended photoperiods associated with summers at high latitudes), flies carrying the *ls-tim* allele are more rhythmic than the *s-tim* flies (note: in *Drosophila*, as in mammals, continuous light is disruptive for the clock) and are able to delay their evening activity under extended photoperiods. Another recent study [11] found that *ls-tim* flies (but not *s*-*tim*) can synchronize their pacemaker to temperature diurnal cycles, which negates the disruptive effect of long photoperiods. The fact that both these behavioral adaptations seem to work best at high latitudes substantiates the putative role of directional selection responsible for the propagation of the *ls-tim* allele across Europe.

A functional molecular polymorphism was also found in *cryptochrome* (*cry*), a gene that in *Drosophila* encodes a blue light circadian-dedicated photoreceptor. *Cry* also serves as an electromagnetic field sensor and as a geotaxis behavior regulator. A recent survey of wild alleles of *cry* [12] revealed a substantial polymorphism, including two major haplogroups, of which one is a 12 bp insertion/deletion polymorphism (B1/B2 alleles). Across Europe, the frequency of B1/B2 alleles correlated with seasonal bioclimatic variables. This spatial distribution of *cry* polymorphisms corroborates the importance of these haplotypes for local adaptation of the clock.

Until recently, studying genetic variation at the genomic level in natural populations was challenging due to the cost of sequencing whole genomes in a large number of individuals. However, the development of new sequencing strategies, such as sequencing DNA from pools of individuals (Pool-Seq [13]) made genome-wide population genetic analysis feasible and paved the way for a new field of population genomics. In a recent study, Kapun et al. [14] carried out the first comprehensive, continent-wide genomic analysis of genetic variation in European populations of *Drosophila melanogaster*. The analysis consisted of 48 Pool-seq samples from 32 population samples collected in 2014 by the European *Drosophila* Population Genomics Consortium (*DrosEU*) [14]. Each pool consisted of 40 flies.

The current study was aimed at analyzing the genetic variation that is encapsulated in the DrosEU dataset and identifying polymorphisms that serve as molecular adaptations of the circadian clock. We identified single nucleotide polymorphisms (SNPs) that show latitudinal clines and tested their functional role. We analyzed eight SNPs in the core clock genes *per*, *tim*, *Clk*, *Dbt*, *and Sgg* and in the output genes *pdf* and *pdfr*. In addition, we analyzed a clinal SNP in the gene *eyes absent* (*eya*), which was recently shown to drive the photoperiodic diapause in *Drosophila* through interaction with TIM [15].

## 2. Materials and Methods

### 2.1. Fly Maintenance

Flies were maintained using standard sugar food at 20 °C under a 12:12 light:dark (LD) cycle. The flies were kept in population cages, which were constructed from 1.5 litter plastic bottles. A 50 mL polypropylene tube containing 30 mL of food was attached to the opening of the bottle. The base of the bottle was made of wire mesh for ventilation of the cage. Sixty-four flies were transferred to each cage using CO_2_ anesthesia (see details below).

### 2.2. Identification of Clinal SNPs

The number of reads supporting each allele at a specific SNP locus in each Pool-seq library was previously counted [14], and the relative abundance of each allele within the pool was then estimated based on the read counts (each pool consisted 40 flies). To test the effect of latitude on the allele frequency of a given SNP, we used a generalized linear model (GLM) with binomial error structure and logit function. The analysis was performed on the raw allele counts. The SNPs in each gene were tested separately, and the significance values were adjusted for multiple testing using the Benjamini–Hochberg method [16]. McFadden’s pseudo-R^2^ measure was used for estimating the variance explained with the logistic regression models. We also used extended models that included ‘longitude’ and ‘altitude’ variables in addition to ‘latitude’. The simple (‘latitude’ only) and extended models were compared using a χ^2^ difference test.

### 2.3. Functional Analysis of Clinal SNPs

#### 2.3.1. Experimental Populations

To test the effects of candidate alleles at a particular SNP on the phenotype, we used the Mendelian randomization (MR) approach [17]. By crossing different fly strains that have the same allele, new strains were generated whose genetic background was completely mixed (randomized). The phenotype of MR strains carrying different alleles of the SNP was then compared. If a difference was found, it was likely due to the SNP, and not due to other loci in the genome. The MR populations were constructed from eight different parental strains, which ensured that the probability of having other loci fixed between the populations was extremely low.

To establish the MR populations, we used strains from the *Drosophila* Genetic Reference Panel (DGRP), which is a community resource of 205 inbred lines derived from a wild population in Raleigh, USA [18]. The genome of these strains has been sequenced, identifying 4,853,802 (SNPs) and 1,296,080 insertions or deletions (indel) variants. We searched for candidate SNPs using the DGRP2 genome browser [19]. For each SNP, four independent populations were generated, of which two carried the reference allele (called set A and set B) and two sets fixed for the alternate allele (set C and set D). Each set was constructed of eight different DGRP strains, using four females and four males from each.

#### 2.3.2. Locomotor Activity Analysis

The locomotor activity behavior of male offspring from each of the MR populations was analyzed using *Drosophila* Activity Monitors (TriKinetics system, Waltham, MA, USA) as previously described [20]. Adhering to previous studies, only male flies were used since the egg-laying activity of females may mask the circadian system output [21].

Flies were held at 20 °C for 8 days under LD 12:12 h, followed by 8 days of continuous darkness (DD). The data were analyzed using the TriKinetics software and the BioDare2 web server [22]. The free-running period (FRP) and the acrophase (phase in DD) were calculated using the MESA method, where the averaged phase was determined from the wave fit. We excluded flies with an FRP (free-running period) shorter than 20 h and longer than 30 h from the analysis. This filtering was performed because these flies did not exhibit a robust circadian rhythm and were flagged using the analysis software as potential outliers. In total, 90 flies were excluded from the analysis out of the initial sample size of 861. The statistical analysis of the FRP was carried out using a mixed model analysis of variance (ANOVA) with ‘Allele’ and ‘Set’ as the fixed and random effects, respectively. The analysis of the phase data was carried out using circular ANOVA with the ‘aov.circular’ R package (using the LRT method).

#### 2.3.3. Diapause Response

To analyze ovarian diapause, females were collected within 7 h after eclosion, a period during which ovaries are previtellogenic [18]. Diapause was induced by placing the flies in plastic vials at 12 °C for two weeks for a long photoperiod (16 h) and a short one (8 h). A group of control flies, in which the vials were covered with aluminum foil, was also included. After two weeks, the female ovaries were dissected in Dulbecco’s phosphate-buffered saline (PBS), and the diapause status of the female was determined as previously described [23].

#### 2.3.4. Chill Coma Recovery

Emerging flies were maintained at LD 12:12 for 3–4 d and were tested for their chill coma recovery time as previously described [24]. Briefly, at ZT 3 (ZT, Zeitgeber time, hours after lights on), the flies were anesthetized using ice and then transferred to tubes, and cotton plugs were used to cover the tube. The flies were kept on ice at 4 °C for 3 h. At ZT 6, the flies were transferred to a small Petri dish at room temperature, their behavior was videotaped, and the recovery time of each fly was determined. Data analysis was carried out using the *Survival* R library to fit Kaplan–Meier curves, and log-rank tests were used to compare the CCR of different genotypes using χ^2^ statistics with one degree of freedom.

## 3. Results

### 3.1. Identifying Clinal SNPs

Analysis of the 11 genes associated with the circadian clock revealed 9776 SNPs, of which 276 showed a significant latitudinal cline (Table 1). The most polymorphic genes were *Pdf* (0.46 SNP/bp) and *cry* (0.15 SNP/bp), while *sgg* and *Pdfr* showed the lowest density (both 0.02). The largest number of SNPs that were clinal were present in *dbt* and *vri*, while *sgg* and *per* harbored the smallest number of clinal SNPs.

**Figure 1:**
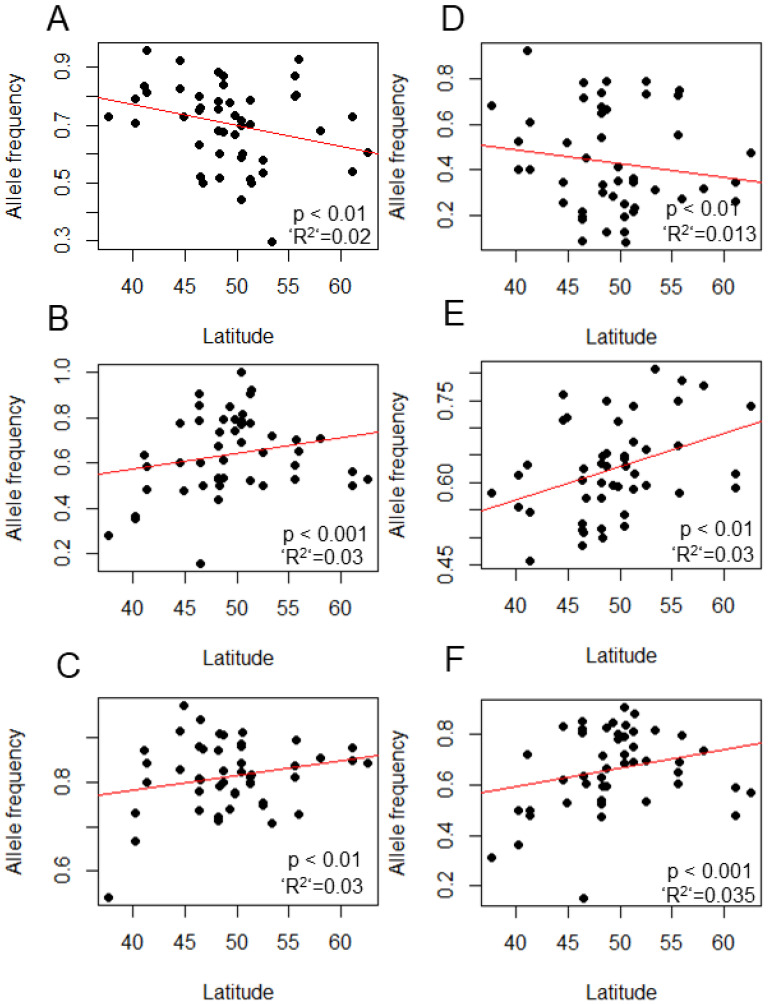
Latitudinal clines in SNPs in clock genes. The frequencies of the reference allele (i.e., the allele that corresponds to the Drosophila v6 reference genome) for six representative SNPs are shown SNPs are shown as data points (filled circles), representing allele frequency measured in pooled DNA sample from each population (n = 40 flies each). The *p*-value for the GLM model and McFadden’s R^2^ are also depicted. The regression line (in red) was drawn using a linear model. (**A**) Pdfr7165A > T, (**B**) sgg3812A > G (**C**) Clk5472G > T, (**D**) per7600A > T, (**E**) Dbt569G > T, and (**F**) sgg3833C > A. Data from reference [11] for samples collected in 2014 (all seasons pooled).

We selected seven SNPs in various clock genes that showed a significant latitudinal cline for further experimental analysis (Table 2). Two other SNPs in *Clk* and *eya* showed a strong correlation with bioclimatic variables in the DrosEU study [10] (but not a latitudinal cline) and were also selected for further analysis. The latitudinal cline in these SNPs is shown in (Figure 1)**.** We also tested the effect of longitude and latitude on the allele distribution using an extended model. The difference between the extended and null (latitude only) models was not significant in Dbt569TG > T (*p* = 0.22), Pdfr7165A > T (*p* = 0.30), tim4740T > A (*p* = 0.22), or Clk5472G > T (*p* = 0.95). In sgg3812A > G, sgg3833C > A, and per7600A > T, the contribution of longitude and altitude was highly significant (*p* < 0.001, for both) and dominated latitude (Supplementary Figure S1).

Previous studies have shown that 19 climatic variables can be summarized using two principal components, PC1 and PC2 [14]. PC1 strongly correlates with latitude, whereas PC2 correlates with longitude to a lesser extent, as well as with latitude and altitude. We conducted a GLM analysis to assess the association between SNP alleles and climatic PC1 and PC2, as presented in Supplementary Table S1. *In SNPs* sgg3812A > G and sgg3833C > A, *there was* a highly significant association with both PCs (McFadden’s R^2^ = 0.16 and 0.11, respectively). In the case of Pdfr7165A > T and Clk5472G > T, a significant association was observed only with PC1 (McFadden’s R^2^ = 0.16 and 0.08, respectively). For per7600A > T, only PC2 exhibited a significant association (McFadden’s R^2^ = 0.34). No significant association was found for eya1849A > G or Dbt569G > T.

### 3.2. Association between SNPs and Circadian Phenotypes

**Figure 2:**
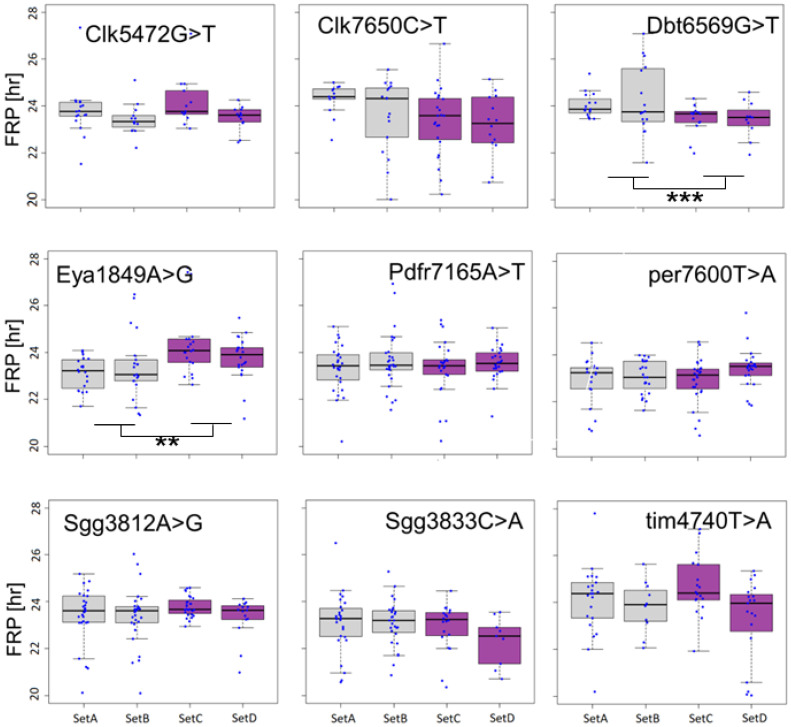
Effect of clinal SNPs on locomotor activity circadian free-running period (FRP). For each SNP, two independent RM populations were tested, fixed for either the reference (box shaded grey) or the alternate allele (purple). In each SNP, the reference allele is the first nucleotide in the SNP ID (i.e., in Eya1849A > G, “A” is the reference allele). The FRP is depicted using a boxplot, where the middle line represents the median, and the box indicates the interquartile range (IQR). The FRP of individual flies (blue dots) is also shown. The error bars represent the 1.5 × IQR interval. ** *p* < 0.01, *** *p* < 0.001.

The selected SNPs were assessed for their impact on circadian phenotypes (note, in the following, we designate each SNP using the last four digits of the genomic position). For each SNP, we compared the circadian free-running period (FRP) between the MR populations that carry different alleles. Two SNPs were significantly associated with FRP variation (Figure 2). In eya1849A > G, the median FRP for the “A” allele (across two sets) was nearly one hour shorter than that of the “G” allele (23.2 vs. 24 h), and the difference was significant (nested ANOVA, F_1,79_ = 9.91, *p* = 0.002). In dbt6569G > T, the median FRP for the “G” allele was nearly 20 min longer (F_1,54_ = 7.5, *p* = 0.008).

**Figure 3:**
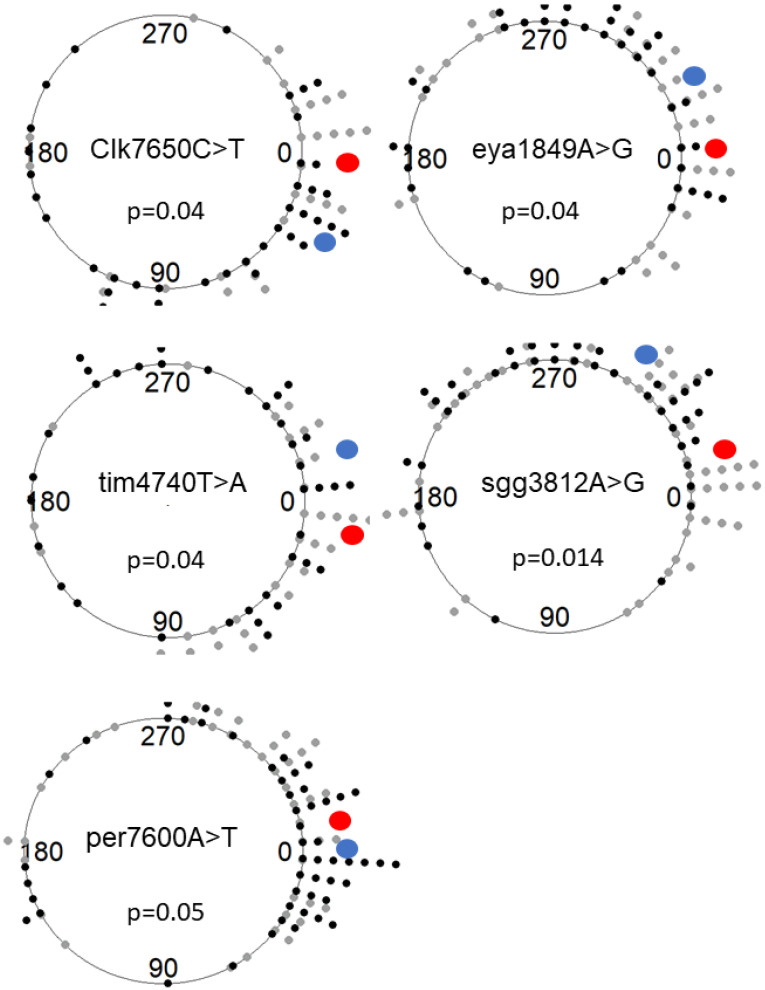
The effect of clinal SNPs on circadian acrophase (phase in DD). The acrophase was calculated over 8 days and depicted with circular plots. Each symbol represents a single observation. Black dots represent the alternate allele, and the gray dots are reference alleles. The red and blue circles represent the circular mean of the reference and the alternate alleles, respectively. The sector between 0° and 180° (bottom of the circle) corresponds to the start (CT0) and end (CT12) of the subjective day (15°  =  1 h), while the sector from 180° to 0° corresponds to the start (CT12) and end (CT24) of the subjective night.

The phase of locomotor activity in DD (acrophase) differed significantly between flies carrying different alleles for five SNPs (Figure 3). These were Clk7650C > T (χ^2^ = 4.10, *p* = 0.043), eya1849A > G (χ^2^ = 4.09, *p* = 0.043), per7600A > T (χ^2^ = 3.80, *p* = 0.051), sgg3812A > G (χ^2^ = 5.96, *p* = 0.014), and tim4740T > A (χ^2^ = 4.16, *p* = 0.041).

### 3.3. Polymorphism in Eya Is Associated with Seasonal Phenotypes

While not recognized as a circadian clock protein, EYA has been implicated in photoperiodism and seasonal timing both in mammals [25,26] and in fruit flies [15]. Therefore, we examined whether the eya1849A > G polymorphism is associated with variation in seasonal response phenotypes. This SNP was previously shown to correlate with a bioclimatic compound variable but not a latitudinal cline [14]. However, GLM analysis revealed significant longitudinal (z = 3.7 *p* < 0.001) and altitudinal (z = 2.8 *p* < 0.005) clines. The frequency of reference allele “A” was higher in the east (where the climate is more continental) and in higher altitudes (Supplementary Figure S1).

**Figure 4:**
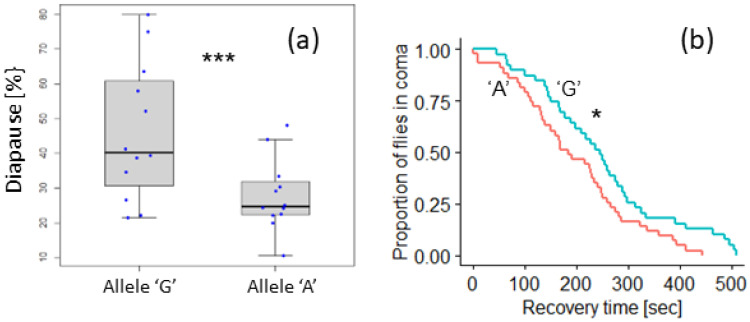
The eya1849A > G polymorphism is associated with the seasonal response. (**a**) Boxplot showing the effect of the *eya* clinal polymorphism on diapause. Proportions of females homozygous for either allele ‘G’ (*n* = 280) or allele ‘A’ (*n* = 359). Data are pooled over replicate sets and photoperiods. In each box, the middle line represents the median, and the box indicates the interquartile range (IQR). The diapause proportion of each sample (blue dot) is also depicted. The error bars represent the 1.5 × IQR. *** *p* < 0.001. (**b**) Chill coma recovery curves showing the chill coma recovery time for flies carrying the ‘A’ allele (red, *n* = 43, set A and set B pooled) and the G allele (blue, *n* = 39, set C and set D pooled). * *p* < 0.05.

Under both short and long photoperiods, developmental arrest of ovaries was significantly higher in females carrying the allele ‘G’ than in females with the ‘A’ allele (median across photoperiod 40% vs. 25%; Figure 4a). The difference was highly significant (binomial general linear model, z = −3.78, *p* < 0.0001).

We also tested the association between the eya1849A > G polymorphism and variation in the cold response using the chill coma recovery assay (Figure 4b). Flies carrying allele ‘A’ were more cold-adapted and recovered significantly faster than flies with the ‘G’ allele (median 177 vs. 243 s; log-rank test, χ^2^ = 4.5, df = 1, *p* = 0.03).

## 4. Discussion

One of the main goals of analyzing genetic variation in wild populations is the potential for identifying local molecular adaptations that are driven by natural selection. Here, we identified 276 SNPs in genes associated with the circadian clock, whose spatial allelic distribution follows a latitudinal cline. We tested the functional role of nine SNPs and found phenotypic effects in seven of them.

In the SNP in *dbt*, the reference allele “G” is present at a higher frequency at higher latitudes. The FRP of flies carrying this allele is longer (Figure 2). Such correlation between FRP and latitude was previously observed in the eclosion rhythm of *Drosophila auraria* [27] and in the leaf movement rhythm in *Arabidopsis thaliana* [28]. It was suggested that longer FRP in the north allows the pacemaker to track the considerable variation in photoperiod that is experienced throughout the year in these regions.

Principal component analysis of 19 bio-climatic variables across Europe revealed two principal components that accounted for 63% of the variation [14]. The allele frequencies of the eya1849A > G polymorphism were associated with the second principal component (PC2), which represented mostly seasonal variables (e.g., isothermality, temperature seasonality). Geographically, PC2 is highly correlated with longitude, reflecting the fact that seasonal fluctuations intensify in Eastern Europe. Notably, we found that the *eya* polymorphism is correlated with longitude, with the “A” allele being more frequent in the east. This allele was also more abundant at higher altitudes. The enhanced cold resistance of this allele, as demonstrated using the CCR (Figure 4), is congruent with its geographical distribution. Intriguingly, the “A” allele confers lower diapause propensity compared to females carrying the “G” allele. Previous studies showed that the CCR and diapause phenotypes are often not correlated and may represent different adaptation mechanisms [24,29]. The SNP in *eya* was also associated with the FRP (Figure 2), but given the longitudinal cline in this polymorphism, its adaptive role is unlikely to be related to the photoperiod and awaits further investigation.

We also found a longitudinal cline in the two SNPs in *sgg* and in *per*, which surpassed their latitudinal cline. Given the correlation between longitude and seasonal bio-climatic variables in Europe, we expect that alleles sgg3812A, sgg3833C, and per7600T, which are more abundant in the east, would be associated with cold adaptations. Interestingly, previous artificial selection experiments using *Drosophila* identified a quantitative trait locus in *sgg* that was associated with increased thermal performance and exhibited a clinal variation in wild populations [30]. Overall, the longitudinal clines that we identified underscore the importance of this climatic gradient in the Eurasian Palearctic biogeographic zone (longer and colder season in the East compared to the West), which is frequently overlooked compared to latitudinal clines [31].

We acknowledge that while our study found statistically significant clinal SNPs, the coefficient of determination (R2) value associated with their effect was low, indicating that the models do not explain much of the variability in the data. This suggests that there are likely other factors at play that we did not measure, or that the spatial distribution of the alleles being studied is shaped with more complex intricate processes. Despite these limitations, it is important to consider the broader implications of our findings. The small clinal effects observed across numerous wild populations of Drosophila in Europe may appear subtle, but they contribute valuable insights into the dynamics of genetic variation and local adaptation. By conducting the first comprehensive survey of single nucleotide polymorphisms (SNPs) in circadian clock genes across multiple populations, our study reveals a nuanced landscape of genetic diversity and adaptation. Furthermore, the identification of associations between SNP alleles and climatic principal components expands our understanding of the complex interplay between genetic variation and environmental factors. Thus, despite the subtlety in the geographical clines and the low R2 values, our study significantly advances the field by providing a comprehensive dataset and shedding light on the genetic dynamics of circadian rhythms in natural populations. We believe that our work makes a valuable contribution by identifying several alleles that mediate phenotypic variation in circadian behavior and seasonal response.

To understand the mechanisms by which these SNPs mediate their phenotypic effects, further experiments will be required, looking at the expression of the different variants at the transcript and protein levels. The phenotypic effect of different alleles may also be manifested by affecting the interaction between their encoded proteins and other clock proteins. For example, the *ls-tim* polymorphism expresses two variants, S-TIM, which binds robustly to CRY, and L-TIM, which exhibits a much weaker interaction [32]. The difference between allelic variants could also affect the transport of proteins between the nucleus and the cytoplasm, which is a significant part of the molecular pacemaker. For example, when flies in the laboratory are maintained under continuous light and temperature diurnal cycles, S-TIM remains cytoplasmic constantly, while L-TIM shows typical nuclear accumulation at ZT0 [11].

Most of the SNPs that were tested here were intronic, alluding to their potential role in gene regulation. Indeed, studies in recent years suggest that variation in non-coding sequences may have an important regulatory role [13]. Introns harbor numerous sites that constitute a functional element, including intron splice enhancers, silencers that regulate alternative splicing, and other regulatory elements. Functional polymorphism within introns can affect gene expression, and intronic variants may modulate the link between genotype and phenotype. Mutations in non-coding DNA may affect remote genes at a distance by altering the splicing efficiency of genes or their transcriptional activity or modulating the expression of the alternative transcripts. Thus, the ‘clock’ SNPs that we found to impact circadian phenotype in this study may mediate their effect via transcriptional regulation.

Several genomic surveys of wild *Drosophila* populations have been carried out in the last decade, and notably, ‘clock’ genes are often present among loci that show substantial genetic differentiation or clinal variation. Polymorphisms in *tim*, *timout*, *Clk*, and *cry*, which were associated with bioclimatic principle components in European populations [14], were also found to be clinal in a survey focusing on North American *Drosophila* populations [33]. A polymorphism in *sgg* was flagged as being associated with climatic variables both in Europe [14] and in another North American survey [34]. Consistently, whole-genome scans for adaptive differentiation identified *sgg* as a significant candidate gene in both European and North American datasets. The fact that the same polymorphisms are clinal on different continents may further indicate their adaptive role. It is noteworthy that all the SNPs that were studied here are segregated in appreciable frequencies in both Europe and North America, as evident from the DGRP strains that were used to generate the MR population in this study. Overall, these studies underscore ‘clock’ genes as a major target for natural selection, which allows rapid adaptation to different climatic conditions.

The immense advance in DNA sequencing technologies in recent years generated an unprecedented amount of data at the genetic level, including genomic sequences and genetic variation, but the functional importance of this variation is largely unknown. A major challenge in current research is to bridge the gap between genomic data and their phenotypic effect by developing high-throughput phenotyping techniques, setting the stage for the new field of ‘phenomics’ [35]. Understandably, the present phenomics screen focus on cellular, morphological phenotypes that are more amenable to high-throughput recording. The challenge would be high-throughput phenotyping of complex phenotypes such as behavior in whole animals. While recording the locomotor activity of a large number of individuals is feasible using *Drosophila*, our experience from the current work suggests that the main ‘bottleneck’ is the generation of congenic strains that are fixed for different SNP alleles. This process, either carried out using the Mendelian randomization approach or transgenics, is laborious, costly, and time-consuming. Hopefully, new CRISPR transgenesis protocols (e.g., [36]) will help bridge the genomics–phenomics gap.

## 5. Conclusions

We identified a large number of SNPs in circadian clock genes that segregate in European populations and exhibit latitudinal clines. These polymorphisms may represent local molecular adaptions of the circadian system. We tested the functional role of nine SNPs and found phenotypic effects in seven of them. This study adds to previous research toward the understanding of how the clock is fine-tuned to perform in different environments and molecular evolution of the clock genes, an area in which relatively little is known about.

## Figures and Tables

**Table 1:** Molecular polymorphisms in circadian clock genes in European *Drosophila* populations. The analysis is based on 48 Pool-seq libraries (*n* = 40 each).

Genes	Genomic Region	Total SNPs	Clinal SNP
*Clk*	3L: 7,763,233–7,775,603	1172	18
*cry*	3R: 19,212,154–19,215,442	488	37
*cwo*	3R: 10,388,258–10,400,752	751	25
*cyc*	3L: 19,813,770–19,815,938	302	18
*dbt*	3R: 31,054,085–31,061,210	727	49
*Pdf*	3R: 26,455,462–26,456,141	311	18
*Pdfr*	X: 2,552,206–2,578,640	685	18
*per*	X: 2,685,580–2,692,780	283	7
*sgg*	X: 2,633,952–2,679,553	1110	5
*tim*	2L: 3,493,986–3,508,119	1864	36
*vri*	2L: 5,288,944–5,311,223	2083	45

**Table 2:** SNPs selected for functional validation.

Gene	SNP Position	Alleles	Annotation	*p* Value
*Clk*	3L: 7,765,472	G/T	missense_variant	0.02
*Clk*	3L: 7,757,650	C/T	Non-synonymous (missense)	a
*dbt*	3R: 31,056,569	G/T	missense_variant	0.018
*Eya*	2L: 6,511,849	A/G	upstream_gene_variant	a
*Pdfr*	X: 2,577,165	A/T	intron_variant	0.04
*per*	X: 2,687,600	T/A	intron_variant	0.051
*sgg*	X: 2,643,833	C/A	intron_variant	0.03
*sgg*	X: 2,643,812	A/G	upstream_gene_variant	0.03
*tim*	2L: 3,504,740	T/A	intron_variant	0.014

a A SNP showing a significant correlation with bioclimatic variables in the DrosEU data [14].

## Data Availability

All genomic DrosEU data are available on the ‘Drosophila Evolution over Space and Time’ website (https://dest.bio (accessed on 11 June 2023)).

## Supplementary Materials

The following supporting information can be downloaded at: https://www.mdpi.com/article/10.3390/biology12060858/s1, Figure S1: Longitudinal and altitudinal clinal SNPs. Table S1: General Linearized model (GLM) testing the effect of climatic compound variables on SNP allele distribution.
