# Supplementary material for: Unveiling Subtle Geographical Clines: Phenotypic Effects and Dynamics of Circadian Clock Gene Polymorphisms
Source: Biology (Basel). 2023 Jun 14;12(6):858. doi: 10.3390/biology12060858 (PMC10295014; doi:10.3390/biology12060858)
Supplement: Supplementary file 1 [file biology-12-00858-s001.zip › biology-2109479-supplementary.pdf]

Mapping molecular adaptations in circadian clock and seasonal response genes of *Drosophila melanogaster*

Supplementary Material

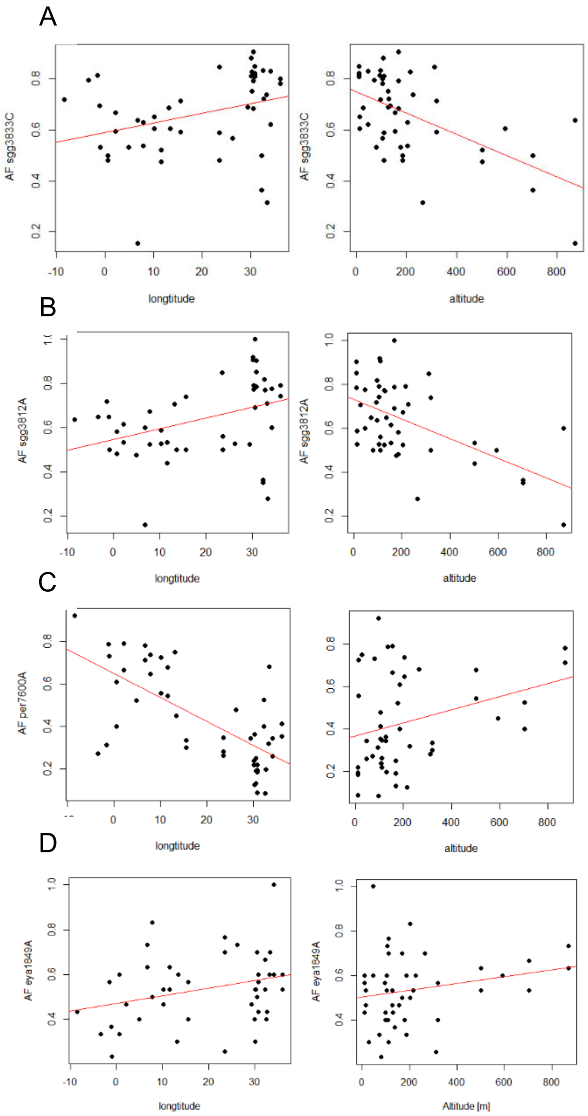

Figure S1. Longitudinal and altitudinal clinal SNPs. The allele frequencies (AF) of the reference allele of four SNP are shown. The regression line was drawn using a linear model. **A** sgg3833C (longtitude: left, altitude: right), **B** sgg3812A **C** per7600A, **D** eya1849A. Data from Kapun et al 2020 (doi:10.1093/molbev/msaa120), of samples collected in 2014 (all seasons pooled).

Table S1. General Linearized model (GLM) testing the effect of climatic compound variables PC1 and PC2 on SNP allele distribution.

sgg3812A>G

	Estimate	Std. Error	z value	Pr(>|z|)	
**(Intercept)**	0.63128	0.05816	10.854	< 2e-16	***
**pc1**	-0.05281	0.01912	-2.762	0.00574	**
**pc2**	-0.16601	0.02423	-6.851	7.32e-12	***

McFadden's pseudo $R^2 = 0.16$

sgg3833C>A

	Estimate	Std. Error	z value	Pr(>|z|)	
**(Intercept)**	0.72996	0.05582	13.078	< 2e-16	***
**pc1**	-0.05271	0.01885	-2.796	0.00517	**
**pc2**	-0.11936	0.02270	-5.258	1.46e-07	***

McFadden's pseudo $R^2 = 0.11$

per7600A>T

	Estimate	Std. Error	z value	Pr(>|z|)	
**(Intercept)**	-0.24516	0.05298	-4.627	3.71e-06	***
**pc1**	0.01294	0.01813	0.713	0.476	
**pc2**	0.30257	0.02315	13.069	< 2e-16	***

McFadden's pseudo $R^2 = 0.34$

Dbt569G>T

	Estimate	Std. Error	z value	Pr(>|z|)	
**(Intercept)**	0.544084	0.046693	11.652	<2e-16	***
**pc1**	-0.025727	0.017477	-1.472	0.141	
**pc2**	0.009148	0.018954	0.483	0.629	

Pdfr7165A>T

	Estimate	Std. Error	z value	Pr(>|z|)	
**(Intercept)**	0.85422	0.05457	15.653	< 2e-16	***
**pc1**	0.06442	0.02087	3.087	0.00202	**
**pc2**	-0.01643	0.02169	-0.758	0.44867	

eya1849A>G

	Estimate	Std. Error	z value	Pr(>|z|)	
**(Intercept)**	0.146110	0.052850	2.765	0.0057	**
**pc1**	-0.008232	0.018795	-0.438	0.6614	
**pc2**	-0.008000	0.021425	-0.373	0.7088	

Clk5472G>T

	Estimate	Std. Error	z value	Pr(>|z|)	
**(Intercept)**	1.46839	0.04777	30.739	< 2e-16	***
**pc1**	-0.07641	0.01520	-5.026	5.01e-07	***
**pc2**	-0.02645	0.01954	-1.353	0.176	

McFadden's pseudo R^2 = 0.08
